# Inflammation and Microbial Translocation Correlate with Reduced MAIT Cells in People with HIV

**DOI:** 10.20411/pai.v10i1.746

**Published:** 2024-11-21

**Authors:** Angela Ryu, Brian M. Clagett, Michael L. Freeman

**Affiliations:** 1 Rustbelt Center for AIDS Research, Division of Infectious Diseases and HIV Medicine, Department of Medicine, Case Western Reserve University/University Hospitals Cleveland Medical Center, Cleveland, OH; 2 Center for Global Health and Diseases, Department of Pathology, Case Western Reserve University, Cleveland, OH

**Keywords:** MAIT cells, HIV, Inflammation, Microbial translocation, IL-6, Tocilizumab

## Abstract

**Background:**

Optimal control of microbial infections requires mucosal-associated invariant T (MAIT) cells. People living with HIV (PWH) on antiretroviral therapy (ART) can be divided into 2 groups: immune responders (IR) who recover or retain CD4 T cell numbers, and immune non-responders (INR) who do not. Compared to IR, INR have fewer MAIT cells and increased systemic inflammation and microbial translocation, but how these factors affect MAIT cells is unknown.

**Methods:**

MAIT cells from IR, INR, and from controls without HIV were enumerated and characterized by flow cytometry. To determine the links among MAIT cells, inflammation, and microbial translocation, the correlations of MAIT cell numbers to previously published soluble inflammatory markers and plasma microbial genetic sequences were assessed by Spearman analysis. *In vitro* assays were used to support our findings.

**Results:**

MAIT cell numbers were significantly negatively correlated with levels of IL-6 and IP-10 (markers of inflammation); CD14, LPS, and FABP2 (markers of microbial translocation); and with abundance of *Serratia* and other Proteobacteria genetic sequences in plasma. In a separate analysis of PWH on ART receiving the IL-6 receptor antagonist tocilizumab (TCZ), we found that blocking IL-6 signaling with TCZ increased IL-7 receptor expression on MAIT cells and reduced plasma IL-7 levels, consistent with improved uptake of IL-7 *in vivo*.

**Conclusions:**

Our findings suggest inflammation and microbial translocation in PWH on ART lead to a loss of MAIT cells via impaired IL-7 responsiveness, resulting in further increased microbial translocation and inflammation.

## INTRODUCTION

Mucosal-associated invariant T (MAIT) cells are a recently characterized subset of thymus-derived CD3^+^ cells that have characteristics of innate immune cells, including rapid cytokine and lytic granule release and highly conserved specificity [1–3]. MAIT cells exhibit a characteristic restricted T cell receptor (TCR) that recognizes riboflavin (vitamin B2) biosynthesis intermediates presented in the context of the ubiquitously expressed major histocompatibility complex (MHC) class I-related (MR1) protein [4, 5]. As many bacteria and yeasts (but not mammals) synthesize riboflavin [6], this reactivity confers MAIT cell specificity for the microbial metabolome. In humans, MAIT cells are often characterized by their expression of the semi-invariant TCR variable α-chain 7.2 (Vα7.2)-Jα33 and by their high surface expression of CD8 (including CD8αα homodimers), the C-type lectin-like receptor CD161, the interleukin-18 receptor (IL-18R), and dipeptidyl peptidase-4 (DPP4), also known as CD26 [7, 8]. MAIT cells are highly abundant in humans, comprising up to 10% of all CD3^+^ T cells in the circulation and are further enriched at tissue sites such as the gut and liver [9, 10]. This remarkable abundance in humans and conservation across species suggests that MAIT cell functionality is tremendously important for proper immune activity and homeostasis. MAIT cell relevance to human health has been confirmed in 2 reports that identified individuals lacking circulating MAIT cells who had histories of difficult-to-treat viral and/or bacterial infections [11, 12].

In human immunodeficiency virus (HIV) infections, numbers of peripheral blood CD161^hi-^ Vα7.2^+^ MAIT cells are rapidly reduced and do not fully recover in people living with HIV (PWH) even after years of virus suppression with combination anti-retroviral therapy (ART) [13–17]. The mechanisms driving this loss are still unclear, but they may be related to chronic inflammation and/or the translocation of microbial products from the gut to the bloodstream. PWH on long-term ART can be broadly divided into 2 groups: immune responders (IRs) and immune non-responders (INRs). IRs are those who recover or retain peripheral blood CD4 T cell counts ≥500 cell/ml, a condition also known as immune success. INRs, on the other hand, have CD4 T cell counts below 350 cells/ml, even after 2 years or more on ART, a condition sometimes termed immune failure. Because these low CD4 T cell counts are often coupled with elevated CD8 T cell counts, INRs tend to have lower CD4/CD8 ratios than do IRs, and low CD4/CD8 ratios are associated with increased risk of comorbidities and mortality in treated HIV infection [18, 19]. Additionally, in comparison to IRs, INRs have elevated levels of plasma indices of inflammation and microbial translocation, dysregulation of the gut microbiome, and increased T cell activation with evidence for mitochondrial dysfunction in T cells.

We previously characterized circulating MAIT cell frequency and function in IR, INR, and people without HIV (PWoH) [17]. Like others have observed [13–16], we found that CD161^hi^Vα7.2^+^ MAIT cells were proportionally decreased among CD3^+^ T cells in PWH. However, our original study was limited in that we characterized MAIT cells by just CD161 and Vα7.2 expression and did not use MR1/5-OP-RU tetramers which have been shown to accurately label MAIT cells [8, 20], nor did we characterize expression of other phenotypic parameters such as CD26 or CD8αα homodimers. Furthermore, we did not investigate what factors might contribute to the reduced MAIT cell compartment in INR PWH.

In the present study, we analyzed MAIT cells from the Cleveland Immune Failure (CLIF) study [21], a cohort of IR, INR, and PWoH who have been previously characterized for their plasma levels of inflammatory markers [21–23] and microbial nucleic acid transcripts [24], to investigate the associations of MAIT cell numbers (using MR1/5-OP-RU tetramers) and phenotypes (eg, CD161, CD26) with soluble markers of inflammation and microbial translocation. Furthermore, we used samples from a recent clinical trial of administration of the IL-6 receptor (IL-6R) antagonist tocilizumab in PWH [25] to investigate the role of IL-6 signaling on MAIT cells. The goal of our study was to clarify the factors involved in MAIT cell regulation in PWH and to identify potential targets for future interventions to restore MAIT cell activity in PWH.

## METHODS

### Participants and Sex as a Biological Variable

This study utilized cryopreserved peripheral blood mononuclear cells (PBMC) from 3 cohorts of donors. First, specimens were used from IR (n=11, 47% female sex), INR (n=30, 45%), and PWoH controls (n=17, 17%) as part of CLIF study [21]. The CLIF study was an analysis of IR (CD4 T cell counts greater than 500 cells/μL after at least 2 years of virologic control) and INR (CD4 T cell counts less than 350 cells/μL after 2 years of virologic control), and PWoH controls. Table 1 shows CLIF study participant demographics at time of sample acquisition. Second, specimens were used from a randomized, placebo-controlled, crossover trial of administration of the IL-6R antagonist tocilizumab (TCZ) in 31 ART-treated PWH (NCT02049437) [25]. From that study, we utilized specimens from 10 participants from the placebo arm (20% female) and 10 participants from the TCZ arm (30% female), prior to washout and crossover. Baseline demographics of the TCZ study participants are shown in Table 2. Third, specimens from additional PWoH (n=23, 43% female) were used for *in vitro* assays. Due to the fairly low number of female donors available, we did not directly compare results from males and females.

**Table 1.** Characteristics of Cleveland Immune Failure (CLIF) Study Participants at Time of Sample Acquisition

	C	IR	INR
N (female sex at birth, %)	17 (47)	11 (45)	30 (17)
Race/Ethnicity, N (%)			
Black, Non-Hispanic	2 (12)	4 (36)	9 (30)
White, Non-Hispanic	14 (82)	7 (64)	18 (60)
Hispanic, Any race	0 (0)	0 (0)	2 (7)
Unknown/other	1 (6)	0 (0)	1 (3)
Age, years^1^	37 (29-42)	43 (39-49)	47 (42-52)
Peak viremia, HIV-1 RNA copies/mL^1^	NA	193407 (103004-551513)^2^	143500 (19896-406230)^3^
Nadir CD4 T count, cells/ml^1^	NA	150 (35-278)^4^	100 (16-161)^5^
Time on ART, years^1^	NA	10.93 (8.45-12.20)^6^	6.08 (3.39-10.90)^4^
CD4 T count, cells/ml^1^	969 (857-1074)	840 (702-1006)	279 (227-303)
CD8 T count, cells/ml^1^	440 (353-732)	903 (845-1188)	618 (482-799)
CD4:CD8 ratio^1^	2.04 (1.36-2.58)	0.87 (0.73-1.08)	0.39 (0.31-0.60)

**Abbreviations:** C, controls; IR, immune responders; INR, immune non-responders; NA, not applicable.

1 median (IQR);

2 3 missing values;

3 10 missing values;

4 4 missing values;

5 5 missing values;

6 1 missing value

**Table 2.** Baseline Characteristics of Tocilizumab Administration Clinical Trial Participants

	Placebo	Tocilizumab
N (female sex at birth, %)	10 (20)	10 (30)
Race/Ethnicity, N (%)		
Black, Non-Hispanic	5 (50)	9 (90)
White, Non-Hispanic	5 (50)	1 (90)
Hispanic, Any race	0 (0)	0 (0)
Unknown/other	0 (0)	0 (0)
Age, years^1^	46.5 (45-54)	48.5 (45-54)
Peak viremia, HIV-1 RNA copies/mL^1^	72392 (6895-133500)	111098 (29841-141801)
Nadir CD4 T count, cells/ml^1^	252 (207.3-295.3)	197 (114.5-257)
Time on ART, years^1^	13.46 (9.69-20.33)	12.25 (6.48-23.04)
CD4 T count, cells/ml^1^	794 (498.5-875.5)	714.5 (561.8-811.3)
CD8 T count, cells/ml^1^	865.5 (667.5-1222.5)	777.5 (691.5-1215.5)
CD4:CD8 ratio^1^	0.78 (0.73-1.11)	0.85 (0.60-1.03)

1 median (IQR).

### MAIT Cell Characterization by Flow Cytometry

PBMCs were thawed, washed in complete RPMI media (10% fetal bovine serum, 1% L-glutamine, 1% penicillin/streptomycin), incubated with LIVE/DEAD viability dye (Life Technologies), labeled with PE-conjugated MAIT cell-binding MR1/5-OP-RU or negative control MR1/6-FP tetramers (NIH Tetramer Core Facility), and stained with antibodies to CD3 (BUV737, clone SK7; BD), CD4 (BUV395, SK3; BD), CD8α (BV605, SK1; BD), CD8β (BV786, 2ST8.5H7; BD), CD161 (APC, DX12; BD), CD26 (PerCP-Cy5.5, BA5b; BioLegend), TCR Vα7.2 (PE-Cy7, 3C10; BioLegend), CD126 (BV421, M5, BD), and CD127 (PE-Dazzle594, A019D5; BioLegend). Samples were then fixed in 1% paraformaldehyde before acquisition on an LSR-Fortessa flow cytometer (BD). Data analysis was performed using FlowJo v10 (BD). For intracellular analyses, following surface Ab labeling, cells were permeabilized using Cytofix/Cytoperm (BD) then stained with anti-PLZF (AF488, Mags.21F7; ThermoFisher). To enumerate cell counts per μL of peripheral blood, the proportion of viable lymphocytes in the appropriate flow cytometry gate was multiplied by the calculated absolute lymphocyte count provided by the clinical complete blood count with differential.

### Soluble Analyte Analyses

Quantification of D-dimer, soluble (s)CD14, fatty acid binding protein-2 (FABP2; also known as intestinal FABP or I-FABP), IL-6, IL-7, IL-18, interferon-inducible protein-10 (IP-10), hyaluronic acid (HA), lipopolysaccharide (LPS; also known as endotoxin) in plasmas from donors in the CLIF study and in NCT02049437 was performed by enzyme-linked immunosorbent assay (ELISA), Luminex assay, ELLA (ProteinSimple), or MesoScale Discovery assay as described and published previously [21–24]. Metabolic profiling (Metabolon) and reference standardization was used to determine plasma concentrations of p-cresol sulfate (PCS) and indoxyl sulfate (IS) as described and published previously [23].

### Plasma Microbiome Analyses

Microbiomes (as transcripts per million [TPM] reads) from CLIF donors were acquired from nucleic acid sequencing and analyses described previously [24] and deposited in the Gene Expression Omnibus at GSE172557. Plasma microbiome species abundance was aggregated at the genera level and expressed as log(TPM+1). Microbial transcripts matched to 535 bacterial genera of which 346 (64.7%) could be detected in plasma of at least one CLIF donor. The NCBI taxonomy database browser was applied via the “phyloT v2” website to generate newick taxonomy tree files.

### *In Vitro* Stimulation

PBMCs from PWoH were cultured with recombinant human IL-6 (R&D) at 5.0, 50, or 500ng/mL; plate bound anti-CD3 (BD) and soluble anti-CD28 (CD28.2; BD) each at 5mg/mL; or recombinant human IL-12 (BioLegend) and IL-18 (R&D) each at 50ng/mL. After 3d of culture, cells were harvested, incubated with FAM-YVAD-FMK (Active Caspase-1 staining kit, Abcam) or medium control for 1 hour at 37°C, then washed and incubated with LIVE/DEAD viability dye, labeled with MR1/5-OP-RU or MR1/6-FP tetramers, and stained with surface antibodies as described above, including anti-CD69 (PE-Dazzle594, FN50; BioLegend). Separately, PBMCs from PWoH were cultured overnight with recombinant human IL-6 (0.5 ng/mL). After culture, cells were harvested, incubated with LIVE/DEAD viability dye, labeled with MR1/5-OP-RU or MR1/6-FP tetramers, and stained with surface antibodies as described above.

### Statistics

Comparisons between 2 independent groups were performed using nonparametric Mann-Whitney U tests. Comparisons between 2 dependent (paired) groups were performed using Wilcoxon matched-pairs signed rank test. Comparisons among 3 or more independent groups were performed using Kruskal-Wallis test with Dunn’s correction for multiple comparisons. Correlation analyses were performed using Spearman analysis. Analysis of MAIT cell subsets was performed using the permutation test with 10,000 iterations with Simplified Presentation of Incredibly Complex Evaluations (SPICE v6.1) software (NIH) [26]. All other statistics were performed using Prism 10 (GraphPad). In all cases, differences were considered significant if the *P* value (or adjusted *P* value when appropriate) was less than 0.05. Correlogram matrices plotting Spearman correlation coefficient (rho) were generated using the corrplot package running under R (v4.3.2) in RStudio (2024.04.1).

### Study Approval

This work was approved by the Institutional Review Board of University Hospitals Cleveland Medical Center (protocols 01-98-55 and 05-14-04). The TCZ clinical trial is registered at NCT02049437. All participants signed informed consent in accordance with the Declaration of Helsinki and agreed to donate their samples for future research.

### Data Availability

All data are available from the corresponding author upon reasonable request.

## RESULTS

### Reduction of MAIT Cells

**Figure 1.**
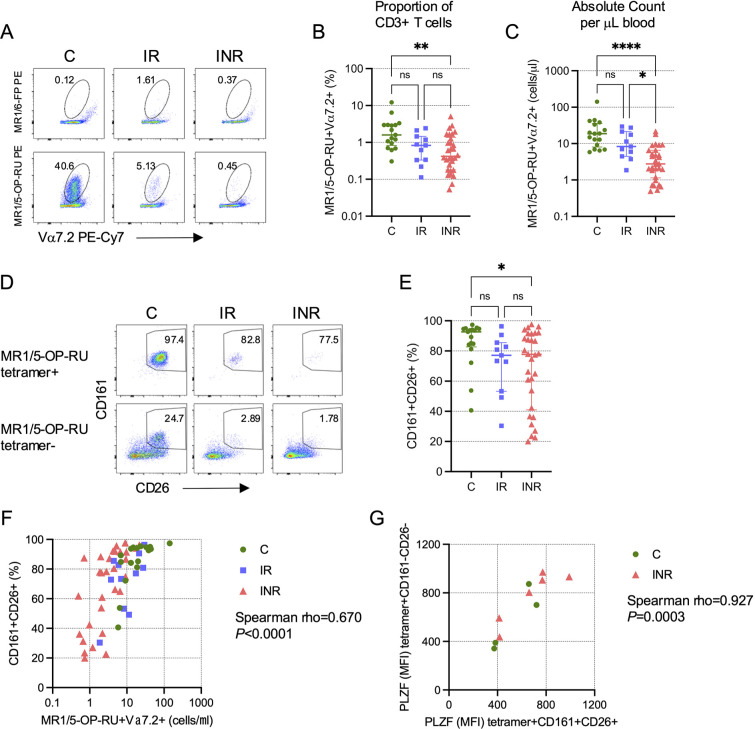
**Reduced MAIT cell proportion and number in PWH with poor CD4 T cell recovery.** Cryopreserved peripheral blood mononuclear cells (PBMCs) from Cleveland Immune Failure (CLIF) study control (C, n=17), immune responder (IR, n=11), and immune non-responder (INR, n=30) donors were thawed, labeled with MR1/5-OP-RU tetramers, and stained with surface antibodies. (A) Representative pseudocolor plots showing binding of PE-conjugated negative control MR1/6-FP (top row) or MAIT cell-binding MR1/5-OP-RU (bottom row) tetramers on Vα7.2^+^CD3^+^ T cells. Numbers on plots indicate the percent of Vα7.2^+^ T cells that bind the indicated tetramer. (B) Proportion of CD3^+^ T cells that are MR1/5-OP-RU^+^Vα7.2^+^. Lines and error bars indicate the median ± IQR. (C) Number of MR1/5-OP-RU tetramer^+^Vα7.2^+^ CD3^+^ T cells per mL of peripheral blood. Lines and error bars indicate the median ± IQR. (D) Representative pseudocolor plots showing CD161 and CD26 expression on Vα7.2^+^CD3^+^ T cells that are MR1/5-OP-RU tetramer^+^ (top row) or MR1/5-OP-RU tetramer^-^ (bottom row). Numbers on plots indicate the percent of cells that are CD161^+^CD26^+^. (E) The percent of MR1/5-OP-RU tetramer^+^Vα7.2^+^CD3^+^ T cells that are CD161^+^CD26^+^. Lines and error bars indicate the median ± IQR. (F) Correlation of the number of MR1/5-OP-RU tetramer^+^Vα7.2^+^CD3^+^ T cells per mL of peripheral blood and the percentage of those cells that are CD161^+^CD26^+^. (G) Correlation of the mean fluorescence intensity (MFI) of intracellular PLZF expression in MR1/5-OP-RU tetramer^+^CD161^+^CD26^+^Vα7.2^+^CD3^+^ T cells and the MFI of PLZF in MR1/5-OP-RU tetramer^+^CD161^-^CD26^-^Vα7.2^+^CD3^+^ T cells in a subset of C (n=4) and INR (n=6) donors. Statistics for B, C, and E were calculated using Kruskal-Wallis test with Dunn’s correction for multiple comparisons. Statistics for F and G were calculated using Spearman correlation. ns, not significant; *P<0.05; **P<0.01; ****P<0.0001.

The CLIF study was a careful investigation of cellular and soluble biomarkers associated with CD4 T cell recovery in PWH on ART [21], but MAIT cell numbers and phenotypes were not included in the initial analyses. We examined MAIT cell numbers in a subset of IR, INR, and PWoH control (C) donors from the CLIF cohort who had viable cryopreserved PBMC samples (Table 1). While the proportion of CD3^+^ T cells that expressed the canonical MAIT cell Vα7.2 TCR (Supplementary Figure 1A) was similar in all groups (Supplementary Figure 1B), the number of Vα7.2^+^ T cells per ml of blood was significantly lower among INR than either IR or C donors (Supplementary Figure 1C). MAIT cells recognize riboflavin (vitamin B2) biosynthesis intermediates presented by the ubiquitously expressed MR1 molecule [4, 5]. Because of this, virtually all MAIT cells can be identified by binding to the MR1/5-OP-RU tetramer [8, 20]. Consistent with our previous study [17], we found that the proportion of CD3^+^ T cells that are MAIT cells (here defined as Vα7.2^+^tetramer^+^) was significantly reduced in INR (Figure 1A,B), and this reduction was even more pronounced when we measured the numbers of Vα7.2^+^tetramer^+^ MAIT cells per ml of blood (Figure 1C).

### Loss of CD161 and CD26 Expression on MAIT Cells in INR

Before the advent of MR1 tetramers, Vα7.2^+^ MAIT cells were often defined by their high co-expression of CD161 and CD26 [7] (Supplementary Figure 2A), and we found a very strong correlation between the proportions of CD3^+^ T cells defined as MAIT cells by MR1/5-OP-RU tetramer binding or CD161^+^CD26^+^ phenotype (rho=0.945, *P*<0.0001; Supplementary Figure 2B). In our previous study, we observed that MAIT cells reduced CD161 expression upon proliferation and that there was an increase in the proportion of Vα7.2^+^CD161^-^ cells that expressed CD8α [17]. We hypothesized then that the reduction in CD161^+^ MAIT cells in INR was not due to the death of Vα7.2^+^ cells, but rather due to a phenotypic conversion precipitated by decreased surface CD161 expression, thereby becoming CD161^-^ “ex-MAIT” cells. We revisited this question here using MR1/5-OP-RU tetramers. Vα7.2^+^tetramer^+^ cells are enriched for a CD161^+^CD26^+^ phenotype (Figure 1D). We posited that “ex-MAIT” cells would maintain the capacity to bind the tetramer even if they lost CD161 expression. While we found no evidence for the accumulation of tetramer-binding cells among the Vα7.2^+^CD161^-^ cells, either proportionally (Supplementary Figure 2C) or as absolute numbers per ml of blood (Supplementary Figure 2D), we did find that Vα7.2^+^tetramer^+^ cells from INR had significantly reduced co-expression of CD161 and CD26 compared to cells from C donors (Figure 1E). Thus, while we did observe a loss of CD161 (and CD26) expression on Vα7.2^+^tetramer^+^ cells in INR donors, this effect did not result in accumulation of Vα7.2^+^tetramer^+^ “ex-MAIT” cells within the CD161^-^CD26^-^ population, suggesting that there is a numeric loss of MAIT cells concurrent with their phenotypic conversion. Consistent with this interpretation, we found that the number of Vα7.2^+^tetramer^+^ MAIT cells was significantly positively correlated with the proportion of these Vα7.2^+^tetramer^+^ MAIT cells that expressed CD161 and CD26 (Figure 1F). Furthermore, Vα7.2^+^tetramer^+^ cells lacking CD161 and CD26 co-expression had similar expression as CD161^+^CD26^+^ cells of the transcription factor promyelocytic leukemia zinc finger (PLZF) (Figure 1G), which has been shown to be important for MAIT cell functionality [27].

MAIT cells are enriched for CD8α expression compared to conventional T cells, with a substantial proportion expressing CD8αα homodimers, as opposed to CD8αβ heterodimers [20, 28]. While CD161 and CD26 expression, and even tetramer-binding capacity, could potentially diminish with conversion to an “ex-MAIT” phenotype, it is unlikely that CD8αα homodimer expression would change. To determine if there was accumulation of CD8αα-expressing cells among the Vα7.2^+^tetramer^-^CD161^-^ population, which would be consistent with a conversion of Vα7.2^+^tetramer^+^CD161^hi^ cells toward a Vα7.2^+^tetramer^-^CD161^-^ “ex-MAIT” phenotype, we examined the proportions of Vα7.2^+^tetramer^+^ and Vα7.2^+^tetramer^-^ cells that expressed CD4, CD8αα homodimers, CD8αβ heterodimers, or that lacked either CD4 or CD8 molecules from C, IR, and INR donors (Supplementary Figure 3A). We observed considerable differences in the composition of the cells between and among the groups (Supplementary Figure 3B). However, even though we found a significant increase in the proportion of Vα7.2^+^tetramer^-^ cells from INR that were CD8αα (Supplementary Figure 3C), we did not find a significant increase in their absolute number (Supplementary Figure 3D). Rather, the proportional change was likely the result of substantial decreases in the proportion (Supplementary Figure 3E) and number (Supplementary Figure 3F) of CD4^+^ Vα7.2^+^tetramer^-^ cells in the INR group, which was expected given that INR are defined by their lack of adequate CD4 T cell recovery on ART. Taken together, our data are consistent with our previous finding that MAIT cells in INR lose surface expression of CD161 yet maintain functionality [17], but in contrast to our prior hypothesis, we did not find evidence of a substantial accumulation of “ex-MAIT” cells in INR individuals, supporting our interpretation that MAIT cell numbers are reduced concurrent with their phenotypic conversion.

### Reduction in MAIT Cells is Correlated with Clinical Indices

**Figure 2.**
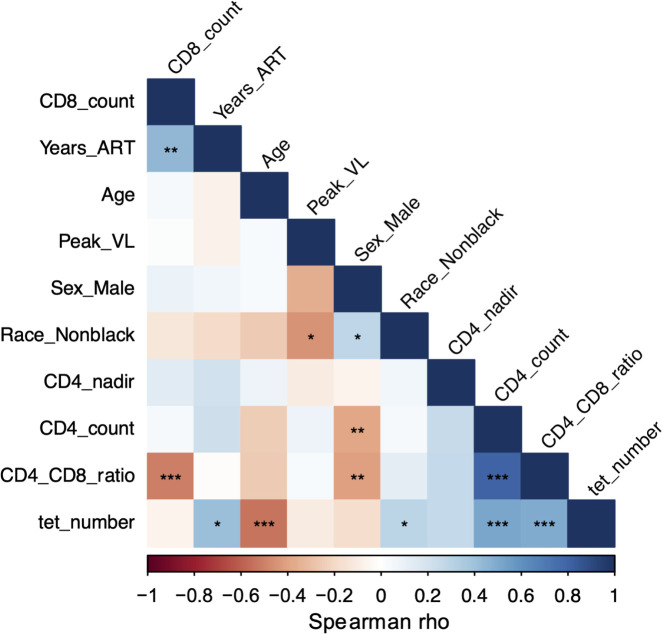
**MAIT cell numbers are negatively associated with age and positively associated with CD4 T cells and CD4/CD8 ratio.** Correlogram of MAIT cell numbers (tet_number) and clinical indices for all available Cleveland Immune Failure (CLIF) donors (n=28-58). Significance calculated using Spearman correlation. *P<0.05; **P<0.01; ***P<0.001.

In the CLIF study [21], INR were more likely white, male, with a lower CD4 T cell nadir, and older at time of ART initiation than IR. In the subset of CLIF participants that we analyzed here, INR were significantly older than C donors but not older than IR donors (Supplementary Figure 4A). As expected, INR had significantly fewer CD4 T cells (Supplementary Figure 4B) and a lower CD4/CD8 T cell ratio than C and IR donors (Supplementary Figure 4C). When comparing HIV-related parameters, there were no significant differences in nadir CD4 T cell count (Supplementary Figure 4D) or peak plasma viral load (Supplementary Figure 4E) between IR and INR, but INR did have significantly shorter time on ART than did IR participants (Supplementary Figure 4F). Moreover, numbers of Vα7.2^+^tetramer^+^ MAIT cells were significantly negatively correlated with participant age, significantly positively correlated with CD4 T cell count, CD4/CD8 ratio, and time on ART, and not associated with CD8 count or peak viral load (Figure 2).

### Reduction in MAIT Cells is Correlated with Soluble Markers of Inflammation and Microbial Translocation

**Figure 3.**
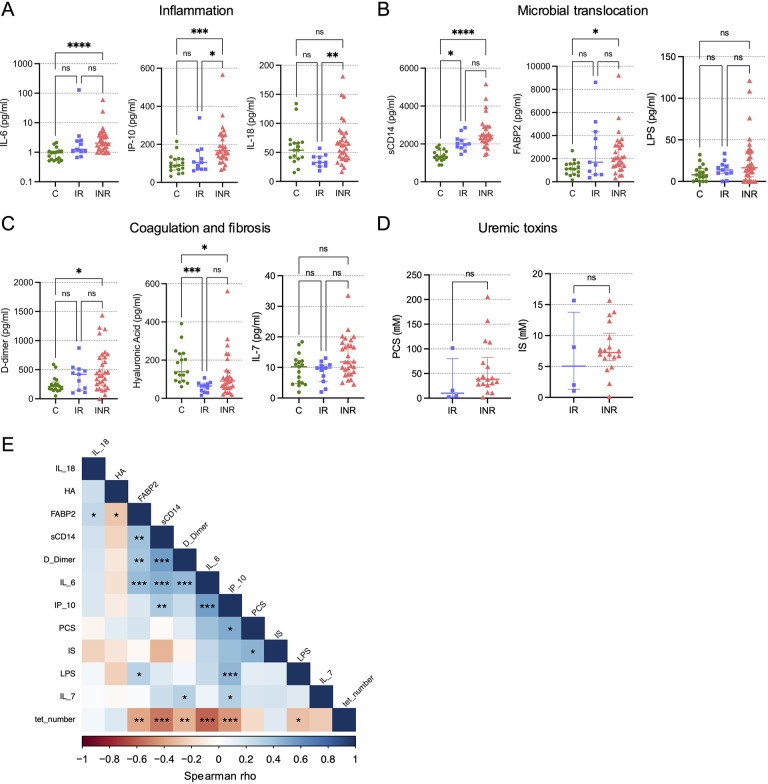
**MAIT cell numbers are negatively associated with soluble markers of inflammation and microbial translocation.** Plasma levels of the soluble markers of inflammation (IL-6, IP-10, and IL-18) (A); microbial translocation (sCD14, FABP2, and LPS) (B); coagulation and fibrosis (D-dimer, hyaluronic acid, and IL-7) (C) were measured for Cleveland Immune Failure (CLIF) study control (C, n=16-17), immune responder (IR, n=10-11), and immune non-responder (INR, n=29-30) donors. Lines and error bars indicate the median ± IQR. (D) Plasma levels of uremic toxins (p-cresol sulfate [PCS] and indoxyl sulfate [IS]) were measured for IR (n=4) and INR (n=18) donors. (E) Correlogram of MAIT cell numbers (tet_number) and soluble plasma analytes for all available CLIF donors (n=22-58). Statistics for A-C were calculated using Kruskal-Wallis test with Dunn’s correction for multiple comparisons. Statistics for D were calculated using Mann-Whitney U test. Statistics for E were calculated using Spearman correlation. *P<0.05; **P<0.01; ***P<0.001; ****P<0.0001.

Plasmas from the CLIF participants have been characterized previously for soluble markers of inflammation, coagulation, and microbial translocation [21–24]. In the subset of CLIF participants studied here, we found that INR had significantly elevated plasma levels of IL-6 and IP-10 (markers of inflammation), D-dimer (a fibrin degradation product associated with mortality in PWH), and sCD14 and FABP2 (markers of microbial translocation) compared to plasma levels in C donors (Figure 3A-C). Levels of IL-18 (a marker of inflammation and inflammasome signaling linked to MAIT cell activity) were significantly elevated in INR versus IR donors, but not compared to C donors. Interestingly, plasma levels of hyaluronic acid (HA), a marker of liver fibrosis [29], were significantly lower in INR and IR compared to levels in plasmas from C donors, which might indicate greater activity of hyaluronidases in PWH than in PWoH. Recently, levels of the bacteria-derived uremic toxins p-cresol sulfate (PCS) and indoxyl sulfate (IS) were shown to be elevated in the plasma of INR compared to IR and to be negatively linked to CD4 T cell counts [23]. While we observed trends toward increased levels of PCS and IS in INR compared to IR (Figure 3D), these differences did not reach statistical significance likely due to the low numbers of donors for whom we had data. Vα7.2^+^tetramer^+^ MAIT cell numbers were significantly negatively correlated with plasma levels of IL-6, IP-10, D-dimer, sCD14, LPS, and FABP2, and many of the soluble analyte levels were positively correlated with each other, suggesting a coordinated inflammatory response linked to a reduction in MAIT cells (Figure 3E). While the relatively low numbers of participants preclude detailed analyses of correlations within each donor group, we did observe consistent trends in-group negative correlations for MAIT cell numbers with IL-6, IP-10, and FABP2 (Supplementary Figure 5).

### Reduction in MAIT Cells is Correlated with Microbial Nucleic Acids in Plasma

**Figure 4.**
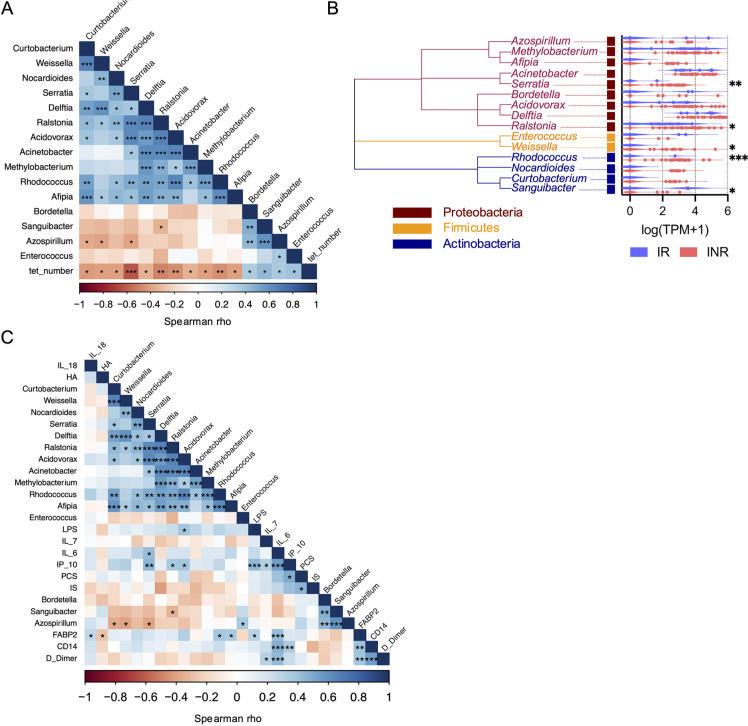
**Increased *Serratia* reads in plasma of INRs are negatively associated with MAIT cell numbers and positively associated with IL-6.** Sequence agnostic shotgun gene deep sequencing of cell-free DNA and RNA fragments in plasma samples from Cleveland Immune Failure (CLIF) study immune responder (IR, n=7) and immune non-responder (INR, n=26) donors was available from GSE172557. Plasma microbiome species abundance (as transcripts per million [TPM] reads) was aggregated at the genera level and expressed as log(TPM+1). (A) Correlogram of MAIT cell numbers (tet_number) and plasma microbiome taxa that were significantly correlated with MAIT cell numbers, for all available CLIF donors (n=33). (B) Phylogenetic tree of plasma microbiome taxa that were significantly correlated with MAIT cell numbers and violin plots of abundance of those microbial elements in IR (n=7) and INR (n=26) donors. (C) Correlogram of plasma microbiome taxa significantly correlated with MAIT cell numbers and soluble plasma analytes for all available CLIF study control (C, n=17, only soluble plasma analytes), IR (n=4-11), and INR (n=18-30) donors. Statistics for A and C were calculated using Spearman correlation. Statistics for B were calculated using Mann-Whitney U test. ns, not significant; *P<0.05; **P<0.01; ***P<0.001.

Due to disrupted gut barrier integrity, PWH experience increased translocation of microbial elements out of the gut to the circulation and other organs that is linked to systemic inflammation [30, 31]. Recently, Nganou-Makamdop et al used sequence agnostic shotgun gene deep sequencing of cell-free DNA and RNA fragments in plasma samples from CLIF donors and other PWH cohorts to demonstrate that the translocated microbiome composition was associated with increased inflammation – particularly IL-6 – and determined immunological outcomes in PWH on ART [24]. We utilized these published analyses to investigate whether certain microbial species were associated with reduced MAIT cell numbers. Of the 535 bacterial genera identified bioinformatically, 346 (64.7%) could be detected in the plasma of at least 1 CLIF donor, and we found the abundance of nucleic acids from 11 genera were significantly negatively correlated with Vα7.2^+^tetramer^+^ MAIT cell numbers in PWH. These were the Proteobacteria genera *Acidovorax, Acinetobacter, Afipia, Delftia, Methylobacterium, Ralstonia*, and *Serratia*; the Actinobacteria genera *Curtobacterium, Nocardiodes*, and *Rhodococcus*; and the Firmicutes genus *Weissella* (Figure 4A,B). Each genus contains species with riboflavin biosynthesis pathways [32, 33], suggesting they could provide direct MR1-restricted antigenic signals to MAIT cells. Vα7.2^+^tetramer^+^ MAIT cell numbers were significantly positively associated with abundance of nucleic acids from 4 bacterial taxa: the Proteobacteria genera *Azospirillum* and *Bordetella*, the Actinobacteria genus *Sanguibacter*, and the Firmicutes genus *Enterococcus* (Figure 4A,B). Compared to IR donors, INR donors had significantly greater amounts of nucleic acids from *Serratia, Ralstonia, Weissella*, and *Rhodococcus*, whereas *Sanguibacter* was significantly more abundant in IR donors (Figure 4B). Interestingly, few inflammatory markers were significantly correlated with bacterial genera that were significantly negatively associated with MAIT cell numbers (Figure 4C), although *Acidovorax* was associated with LPS and *Serratia* was associated with IL-6, an expected result given the known relationships of *Serratia* to IL-6 [24, 34]. *Acidovorax, Ralstonia*, and *Serratia* (all Proteobacteria) were significantly positively correlated with plasma levels of IP-10, as were LPS and sCD14, consistent with an additional link from microbial translocation to type I IFN expression, which has been established previously [30]. Of all the measurements of inflammation and microbial translocation in plasma, only levels of *Serratia* were significantly associated with the proportion of MAIT cells that co-expressed CD161 and CD26 (rho=-0.436, *P*=0.011).

### TCR Signaling, But Not IL-6, Directly Drives MAIT Cell Activation or Death

**Figure 5.**
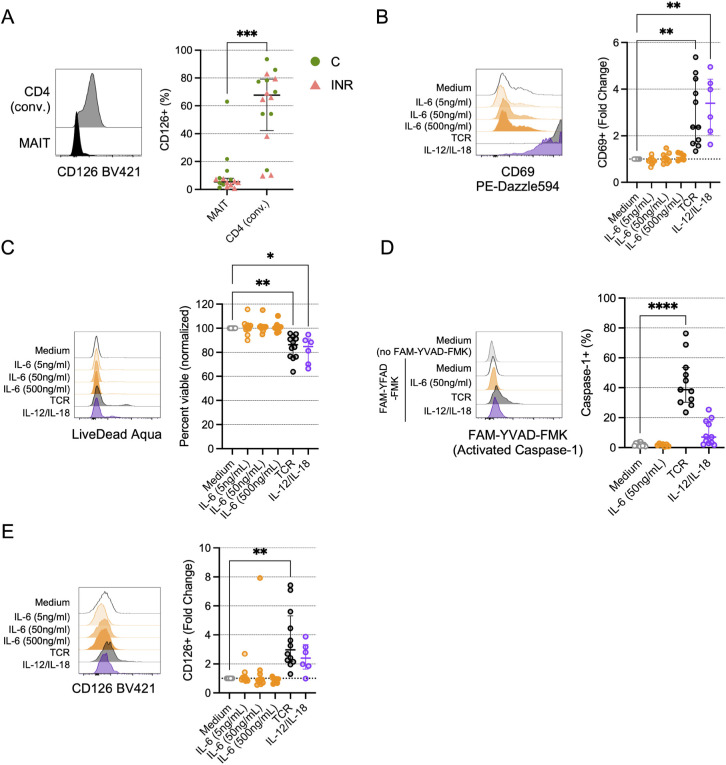
***In vitro* TCR engagement, but not IL-6 exposure, promotes activation and reduces viability of MAIT cells.** Cryopreserved peripheral blood mononuclear cells (PBMCs) from Cleveland Immune Failure (CLIF) study control (C, n=8) and immune non-responder (INR, n=8) donors were thawed, labeled with MR1/5-OP-RU tetramers, and stained with surface antibodies. (A) Representative histograms showing CD126 expression on a C donor (left) and proportion of indicated cells that are CD126^+^ (right) on conventional (conv.) CD4 T cells or MR1/5-OP-RU tetramer^+^Vα7.2^+^ CD3^+^ (MAIT) cells. Lines and error bars indicate the median ± IQR. (B-E) PBMCs from people without HIV (PWoH, n=6-12) were cultured for 3d *in vitro* with indicated concentrations of IL-6, or with anti-CD3 and anti-CD28 (TCR), or IL-12 and IL-18, as described in the Methods. (B) Representative histograms showing CD69 expression (left) and fold-change in CD69 expression over medium control (right) on MAIT cells. Lines and error bars indicate the median ± IQR. (C) Representative histograms showing LIVE/DEAD Aqua accumulation (left) and viability (percent LIVE/DEAD Aqua negative) normalized to medium control (right) on MAIT cells. Lines and error bars indicate the median ± IQR. (D) Representative histograms showing FAM-YVAD-FMK accumulation in MAIT cells (left) and proportion of MAIT cells that accumulated FAM-YVAD-FMK (activated Caspase-1) (right). Lines and error bars indicate the median ± IQR. (E) Representative histograms showing CD126 expression (left) and fold-change in CD126 expression over medium control (right) on MAIT cells. Lines and error bars indicate the median ± IQR. Statistics in A were calculated using Mann-Whitney U test. Statistics for B-E were calculated using Kruskal-Wallis test with Dunn’s correction for multiple comparisons. Only statistically significant differences are labeled. *P<0.05; **P<0.01; ***P<0.001; ****P<0.0001.

Although we found a strong statistical association of MAIT cell numbers with plasma IL-6 levels, it is unclear if IL-6 directly influences MAIT cell viability, phenotype, or function. Compared to conventional CD4 T cells, peripheral blood MAIT cells have low expression of the IL-6R α-chain CD126 (Figure 5A), and IL-6 exposure *in vitro* has little direct effect on MAIT cell CD69 expression [35]. Using cells from PWoH, we found that unlike TCR engagement or IL-12 and IL-18 exposure, stimulation with IL-6 *in vitro* does not lead to MAIT cell activation (Figure 5B) or impaired viability (Figure 5C), nor does IL-6 exposure elicit caspase-1 activation (Figure 5D). Notably, in addition to driving MAIT cell activation and reducing viability, TCR stimulation increased CD126 surface expression (Figure 5E), suggesting that engagement with microbial products *in vivo* could also make MAIT cells more susceptible to IL-6.

### IL-6 Decreases MAIT Cell Expression of the IL-7 Receptor

**Figure 6.**
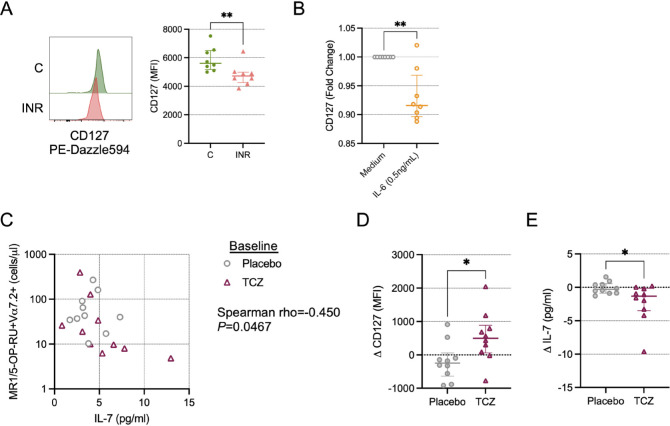
**Inhibition of IL-6 signals *in vivo* restores IL-7 receptor (CD127) expression on MAIT cells.** (A) Cryopreserved peripheral blood mononuclear cells (PBMCs) from Cleveland Immune Failure (CLIF) study control (C, n=8) and immune non-responder (INR, n=8) donors were thawed, labeled with MR1/5-OP-RU tetramers, and stained with surface antibodies. Representative histograms showing CD127 expression (left) and mean fluorescence intensity (MFI) of CD127 staining (right) on MAIT cells. Lines and error bars indicate the median ± IQR. (B) Fold-change in CD127 MFI over medium control on MAIT cells from people without HIV (PWoH, n=8) after overnight culture *in vitro* with IL-6 (0.5ng/mL) or medium control. Lines and error bars indicate the median ± IQR. (C-E) Cryopreserved PBMCs from NCT02049437, from baseline and at week 10 following monthly administrations (at week 0, week 4, and week 8) of tocilizumab (TCZ, n=10) or placebo control (n=10), were thawed, labeled with MR1/5-OP-RU tetramers, and stained with surface antibodies. (C) Correlation of the baseline number of MR1/5-OP-RU tetramer^+^Vα7.2^+^ CD3^+^ T cells per mL of peripheral blood and the baseline concentration of IL-7 in plasma. (D) Change in CD127 MFI from baseline to week 10. Lines and error bars indicate the median ± IQR. (E) Change in plasma IL-7 concentration from baseline to week 10. Lines and error bars indicate the median ± IQR. Statistics in A, B, D, and E were calculated using Mann-Whitney U test. Statistics for C were calculated using Spearman correlation. *P<0.05; **P<0.01.

Plasma levels of IL-7 in PWH are tied to their accessibility and clearance/uptake by cells expressing the IL-7R α-chain CD127 [22, 36]. Thus, higher levels of plasma IL-7 is a sign of more IL-7 production and/or less IL-7 utilization. IL-7 has been shown to rescue antimicrobial functionality of MAIT cells from PWH *in vitro* [37], and IL-7 administration expands MAIT cells in PWH *in vivo* [38]. In the CLIF cohort, we found a negative correlation of MAIT cell numbers with plasma IL-7 levels (Figure 3C), but that did not achieve statistical significance (rho=-0.259; *P*=0.052). Furthermore, in a subset of CLIF participants for whom we measured surface CD127 expression, we found that CD127 levels on Vα7.2^+^tetramer^+^ cells from INR donors were significantly reduced compared to levels on cells from C donors (Figure 6A), consistent with reduced MAIT cell responsiveness to IL-7 in INRs. IL-6 exposure *in vitro* did modestly reduce surface expression of CD127 on MAIT cells from PWoH (Figure 6B), suggesting that *in vivo* IL-6 activity could render MAIT cells less sensitive to IL-7 resulting in reduced numbers and impaired functionality.

Recently, we and our colleagues conducted a randomized, placebo-controlled clinical trial of the IL-6 receptor antagonist tocilizumab (TCZ) in PWH on ART [25]. In that study, we found that plasma levels of several markers of inflammation and homeostasis fell dramatically during TCZ treatment, including sCD14, D-dimer, and IL-7, confirming that IL-6 is a potent pro-inflammatory agent in PWH and blocking its activity with TCZ has a strong anti-inflammatory effect. In a cross-sectional analysis of trial participants receiving placebo or TCZ (n=10 each, Table 2), we found a negative correlation of MAIT cell numbers with plasma IL-7 levels at baseline (rho=-0.450, *P*=0.047, Figure 6C). While blocking IL-6 signaling with TCZ did not significantly increase in MAIT cell numbers or percentages in this small sub-analysis (Supplementary Figure 6), it did result in increased CD127 expression on MAIT cells (Figure 6D), confirming a role for IL-6 in the regulation of MAIT cell responsiveness to IL-7, and a reduction in plasma levels of IL-7 (Figure 6E), consistent with increased uptake of IL-7 *in vivo*. Taken together, our findings suggest a model in which increased microbial translocation and systemic inflammation in INR leads to microbial antigen-specific and antigen-nonspecific activation-induced cell death of circulating MAIT cells that is at least partially mediated by impaired IL-7 signaling.

## DISCUSSION

In this report, we demonstrate a link from plasma microbial nucleic acid transcripts, particularly for those from *Serratia* and other Proteobacteria species, to soluble inflammatory indices and to MAIT cell numbers in PWH. Using MR1/5-OP-RU tetramers, we confirm our previous observations of reduced MAIT cell proportions in INRs compared to IRs and to controls without HIV [17]. We also show that CD161 and CD26 co-expression is reduced on Vα7.2^+^tetramer^+^ MAIT cells in INR, consistent with our previous finding that MAIT cells in INR lose surface expression of CD161. In contrast to our prior hypothesis, however, we find no evidence of a substantial accumulation of “ex-MAIT” cells in INR individuals.

Chronic inflammation (eg, elevated IL-6 and type I IFNs) and microbial translocation from a compromised mucosa are central to the pathogenesis of non-AIDS comorbidities in PWH on suppressive ART [39–41]. It has been hypothesized that MAIT cells are driven to activation-induced cell death (AICD) in viral infections and inflammatory disorders [13, 14, 35, 42–44], but whether this effect is due to antigenic signals (such as via translocation of microbial elements) or to antigen non-specific inflammatory signals is unknown. TCR engagement drives MAIT cell activation and reduces MAIT cell viability. In addition, MAIT cells are exquisitely sensitive to activation via cytokines, including type I IFNs [45, 46], IL-7 [10, 37, 47], IL-15 [48–50], and the combination of IL-12 and IL-18 [49, 51, 52]. Here, we found that MAIT cell numbers were significantly negatively correlated with plasma levels of IL-6 and IP-10 (markers of inflammation); D-dimer (a fibrin degradation product associated with mortality in PWH); and sCD14, LPS, and FABP2 (markers of microbial translocation). Given the plausible role of MAIT cells in promoting mucosal immune homeostasis and gut barrier integrity, and the relationship between microbes and their products and systemic inflammation, it is likely that each of these factors (dysregulated MAIT cells, microbial translocation, inflammatory agents) contributes to the exacerbation of the other factors as well as to morbidities in PWH on ART.

IL-6 is one of the most robust predictors of morbidity and mortality in PWH on ART [53–55]. We and our colleagues recently performed a randomized, placebo-controlled, crossover trial of administration of the IL-6R antagonist TCZ in 31 ART-treated PWH [25]. Results from this trial confirmed that IL-6 is a potent pro-inflammatory agent in PWH and blocking its activity with TCZ has a strong anti-inflammatory effect. Furthermore, TCZ administration reduced plasma levels of inflammatory mediators CRP, sCD14, D-dimer, sTNFR1, sTNFR2, and sCD40L [25]. Therefore, it is plausible that TCZ could have a restorative effect on MAIT cells by alleviating their continued exposure to these and other chronic inflammatory elements. IL-6 may also disrupt immune homeostasis in the gut via interference with IL-7 signals. IL-7 is important for T cell homeostasis and survival [56], and IL-7 has been linked to both MAIT cell numbers and function in PWH [37, 38, 57]. Furthermore, *in vitro* exposure of T cells to IL-6 promotes T cell cycling while inhibiting IL-7 responses at multiple points, such as downregulating the IL-7R α-chain CD127 and impairing IL-7-induced STAT5 phosphorylation and anti-apoptotic Bcl-2 upregulation [36]. Here, we found that IL-6 directly reduced surface expression of CD127 on MAIT cells *in vitro*, and TCZ administration *in vivo* resulted in a significant increase in MAIT cell CD127 expression. Moreover, we found that TCR engagement, which could mimic MR1-mediated activation of MAIT cells by translocated microbial elements, induced upregulation of the IL-6R and could therefore sensitize MAIT cells to IL-6 signals and subsequent impairment of IL-7 responsiveness. Thus, our findings clarify the factors that may be involved in MAIT cell regulation and offer intriguing targets for future interventions to restore MAIT cell activity in PWH.

Where microbes and their products are encountering MAIT and other cells to influence pathogenicity is an open question. Our work here investigated the association of MAIT cells with microbial elements in the peripheral blood, but one limitation of our study is that it is likely that direct microbial/MAIT cell interactions are mostly occurring elsewhere, such as the liver or the gut epithelium, two locations where MAIT cells are highly enriched [9, 10]. Thus, studies that utilize human tissue specimens may allow for more robust characterization of these interactions. Another limitation of our study is that we do not have matching gut microbiome analyses. It is still unclear what selective pressures allow specific gut microbes to access the bloodstream during HIV disease, and having matched peripheral blood and gut microbiome analyses could add insight to the appearance of particular taxa in the plasma, particularly as evidence suggests that gut dysbiosis may influence MAIT cell numbers and function [58, 59]. Additionally, we only compared MAIT cell numbers and plasma microbiota with samples from PWH from a single cross-sectional study, which had a relatively small sample size [24]. Other reports have found differences in the abundance and diversity of the translocated microbiota of PWH and PWoH, as well as in PWH over time on ART [34, 60–62]. Although consistent associations among microbes in plasma and systemic inflammation have been observed [24, 34, 61], whether MAIT cell numbers are linked to *Serratia* or other taxa in these inflammatory settings is unknown, nor is it known whether these associations change over time. Furthermore, we do not distinguish causality – is it the microbial translocation that elicits IL-6 or does inflammation drive gut barrier permeability? On one hand, IL-7 has also been shown to promote IL-17A [47, 63, 64] and IL-22 [65] production in various models. Thus, exposure to increased IL-6 levels could result in mucosal immune cell death and/or failure to express IL-17A and IL-22, which are necessary to maintain an intact gut epithelium [66]. On the other hand, *Serratia* and *Serratia*-derived LPS have been shown to directly elicit IL-6 from PBMCs [24, 34], suggesting that elevated plasma IL-6 is a consequence of microbial elements translocating across the gut epithelium. Determining if and how TCZ administration alters the microbiome and/or microbial translocation will go far to disentangle gut barrier integrity from inflammation. Interestingly, unlike the negative association of MAIT cell numbers with *Serratia*, we found that MAIT cell numbers were positively correlated with the abundance of plasma *Enterococcus* genetic sequences, which were not associated with any of the soluble markers of inflammation. While we did not distinguish taxa at the species level here, Le Bourhis et al showed that *E. faecalis* did not activate MAIT cells *in vitro* [67]. Thus, signals from *Enterococcus* species may not elicit AICD the same way as signals from other translocated microbes and may instead foster MAIT cell accumulation in PWH. A final limitation of our study is that due to the low numbers of available samples, we did not measure MAIT cell functions (such as proliferation or cytokine production) in the same individuals who had their plasma microbiomes characterized. Findings from our previous study were consistent with MAIT cells in INR retaining their capacity to produce IFNγ in response to TCR stimulation, even as those cells lost surface CD161 expression [17], similar to the retention of PLZF expression in Vα7.2^+^tetramer^+^CD161^-^ cells that we observed here. Therefore, we posit that MAIT cell functionality is likely maintained on a per-cell basis in INR, but that there is a reduction in MAIT cell activity on the population level due to the overall decrease in MAIT cell numbers. MAIT cell activity is important for optimal protection from infection in animal models [68], and while preliminary findings regarding MAIT cell activity in humans are consistent with this interpretation [11, 12], data on MAIT cell-mediated protection from infection in humans are scarce. A less appreciated function of MAIT cells is their ability to promote vaccine responses [69, 70]. While some studies have shown PWH to have reduced vaccine responsiveness [71], particularly PWH with low CD4 T cell counts [72–74], no link has yet been established between vaccine efficacy and MAIT cell numbers or function in PWH. It will be important in future studies to determine if vaccine responsiveness is improved in PWH who received TCZ and whether any such improvement is linked to restoration of MAIT cell numbers and/or functionality.

In conclusion, we identify key factors associated with the reduction in MAIT cell numbers in the peripheral blood of PWH with poor CD4 T cell recovery. The combination of antigenic signals, such as those from translocated microbial elements, and inflammatory cytokines like IL-6 leads to the activation and reduction of MAIT cells from the circulation both directly via AICD and indirectly by inhibition of IL-7 signals, respectively. Uncoupling these factors will require further research.

## SUPPLEMENTARY DATA

Supplementary materials are available at the *Pathogens and Immunity* website. Supplementary data may be provided by the authors to benefit the reader. Supplementary data are not copyedited and are the sole responsibility of the authors. Questions or comments related to supplementary materials should be addressed to the corresponding author.
