# Supplementary material for: Inflammation and Microbial Translocation Correlate with Reduced MAIT Cells in People with HIV
Source: Pathog Immun. 2024 Nov 21;10(1):19–46. doi: 10.20411/pai.v10i1.746 (PMC11613984; doi:10.20411/pai.v10i1.746)
Supplement: Supplementary Figures [file pai-10-019-s01.pdf]

PATHOGENS AND IMMUNITY
In Association with Case Western Reserve University and The Richard J. Fasenmyer Foundation

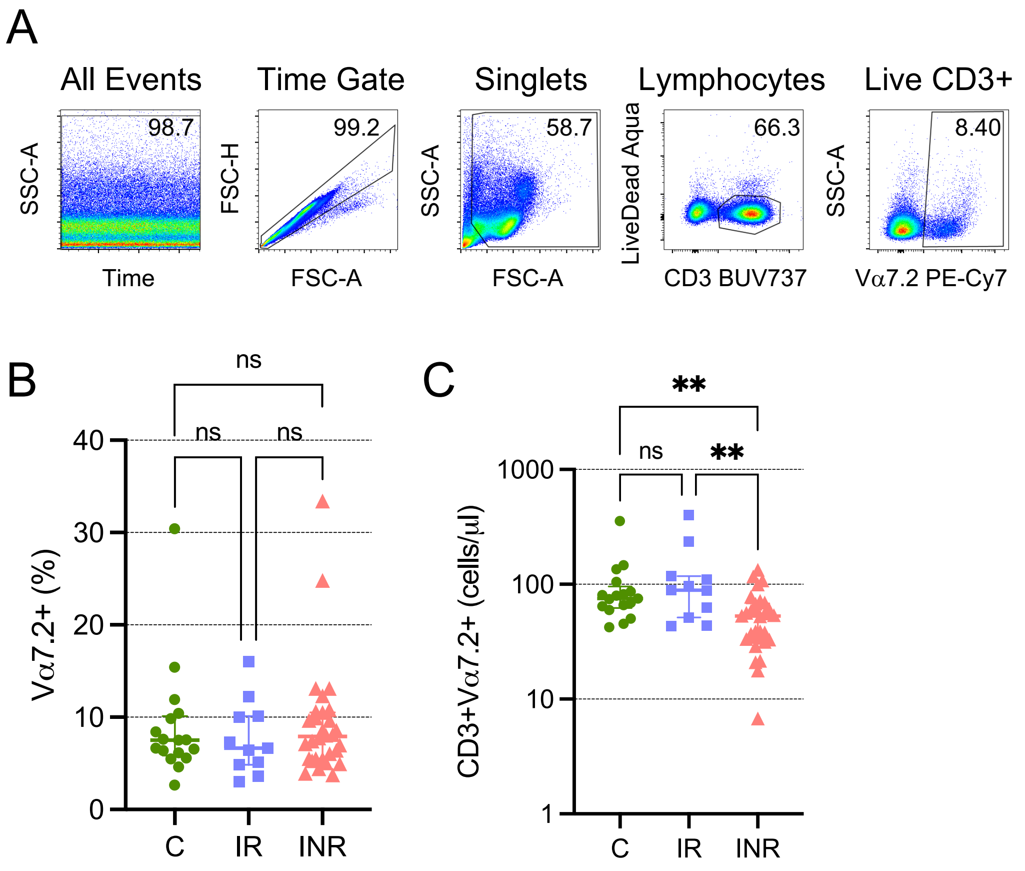

Supplementary Figure 1. Reduced number of TCR Vα7.2+ CD3+ T cells in PWH with poor CD4 T cell recovery. Cryopreserved peripheral blood mononuclear cells (PBMCs) from Cleveland Immune Failure (CLIF) study control (C, n=17), immune responder (IR, n=11), and immune non-responder (INR, n=30) donors were thawed and stained with surface antibodies. (**A**) Representative pseudocolor plots showing gating strategy to identify live CD3+ T cells expressing the Vα7.2 T cell receptor (TCR). Numbers on plots indicate the percent of cells in the indicated gate. (**B**) Proportion of $CD3^+$ T cells that are $V\alpha7.2^+$. Lines and error bars indicate the median ± IQR. (**C**) Number of $V\alpha7.2^+$ $CD3^+$ T cells per μL of peripheral blood. Lines and error bars indicate the median ± IQR. Statistics were calculated using Kruskal-Wallis test with Dunn's correction for multiple comparisons. ns, not significant; $^{**}P<0.01$.

Ryu A, Clagett BM, Freeman ML. Inflammation and Microbial Translocation Correlate with Reduced MAIT Cells in People with HIV. *Pathogens and Immunity*. 2024;10(1):19–46. doi: 10.20411/pai.v10i1.746

PATHOGENS AND IMMUNITY
IN ASSOCIATION WITH CASE WESTERN RESERVE UNIVERSITY AND THE RICHARD J. FASENMYER FOUNDATION

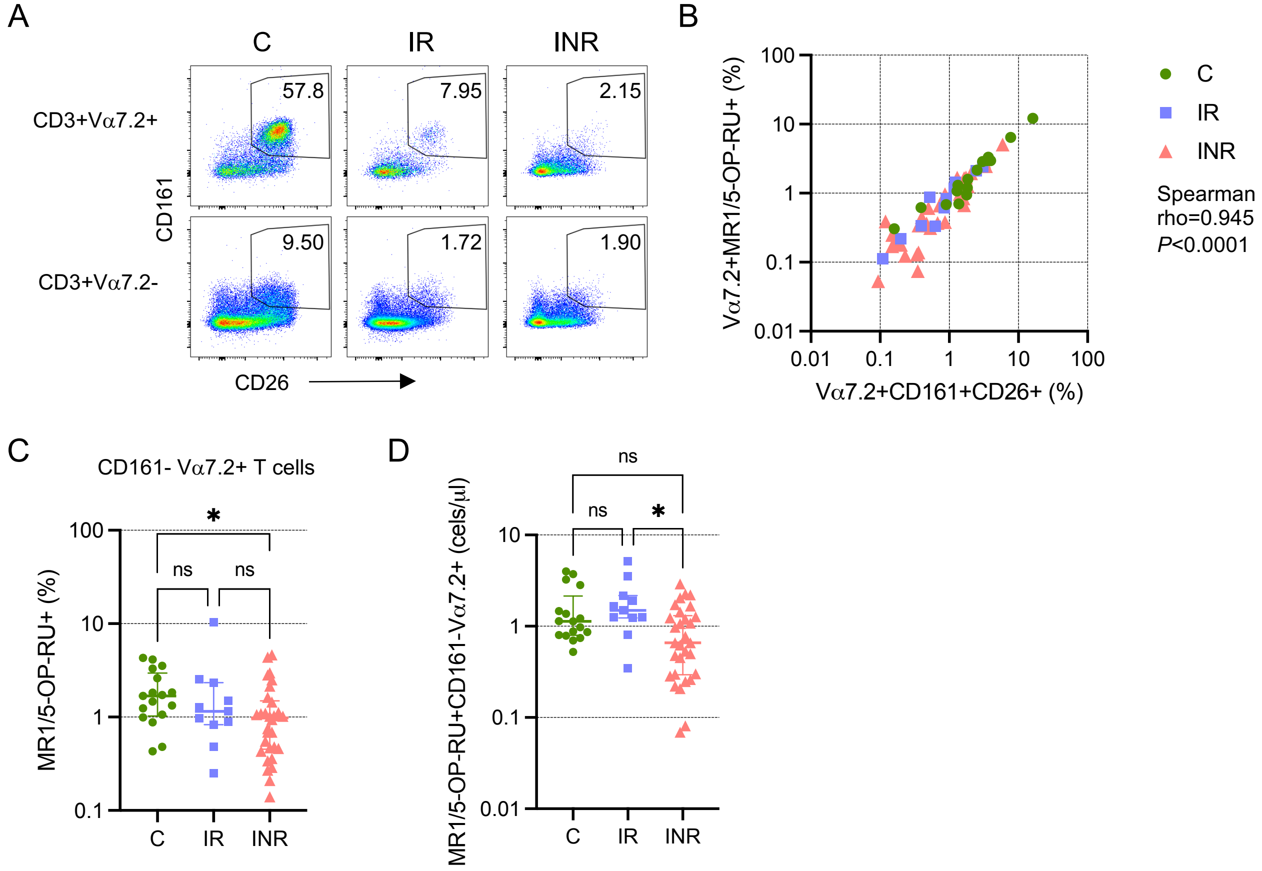

Supplementary Figure 2. There is no accumulation of MR1/5-OP-RU+ cells among Vα7.2+ T cells lacking CD161. Cryopreserved peripheral blood mononuclear cells (PBMCs) from Cleveland Immune Failure (CLIF) study control (C, n=17), immune responder (IR, n=11), and immune non-responder (INR, n=30) donors were thawed, labeled with MR1/5-OP-RU tetramers, and stained with surface antibodies. (**A**) Representative pseudocolor plots showing CD161 and CD26 staining on Vα7.2$^+$ CD3$^+$ T cells (top row) or Vα7.2$^-$ CD3$^+$ T cells (bottom row). Numbers on plots indicate the percent of cells that co-express CD161 and CD26. (**B**) Correlation of the percent of Vα7.2$^+$CD3$^+$ T cells that are CD161$^+$CD26$^+$ and the percentage of Vα7.2$^+$CD3$^+$ T cells that bind the MR1/5-OP-RU tetramer. (**C**) Proportion of Vα7.2$^+$CD161$^-$ CD3$^+$ T cells that are MR1/5-OP-RU tetramer$^+$. Lines and error bars indicate the median ± IQR. (**D**) Number of MR1/5-OP-RU tetramer$^+$Vα7.2$^+$CD161$^-$ CD3$^+$ T cells per μL of peripheral blood. Lines and error bars indicate the median ± IQR. Statistics for **B** were calculated using Spearman correlation. Statistics for **C** and **D** were calculated using Kruskal-Wallis test with Dunn's correction for multiple comparisons. ns, not significant; **P*<0.05.

PATHOGENS AND IMMUNITY
IN ASSOCIATION WITH CASE WESTERN RESERVE UNIVERSITY AND THE RICHARD J. FASENMYER FOUNDATION

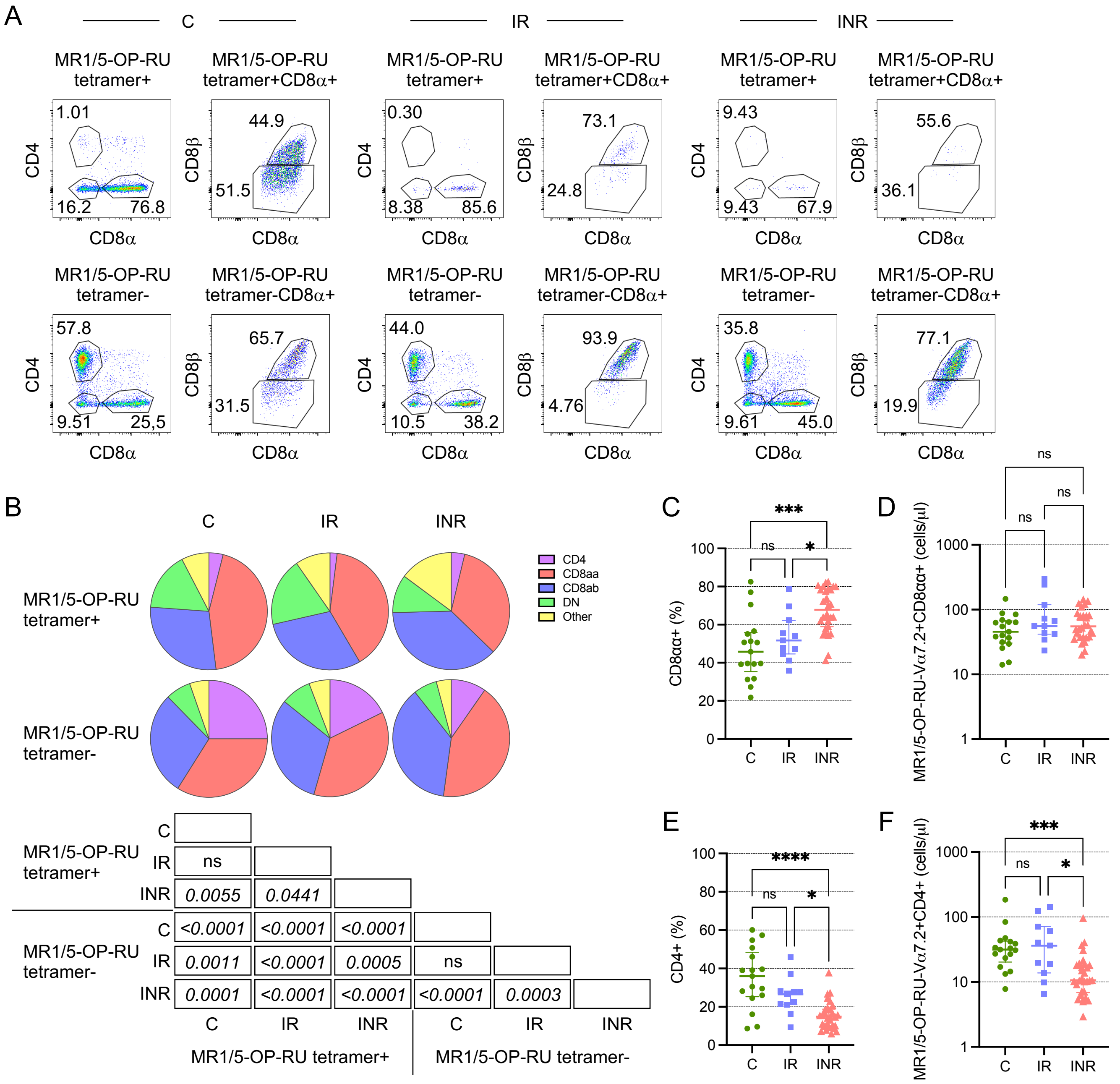

Supplementary Figure 3. Reduced CD4+ cells in MAIT cells from INR drives the proportional increase in CD8+ MAIT cell subpopulations. Cryopreserved peripheral blood mononuclear cells (PBMCs) from Cleveland Immune Failure (CLIF) study control (C, n=17), immune responder (IR, n=11), and immune non-responder (INR, n=30) donors were thawed, labeled with MR1/5-OP-RU tetramers, and stained with surface antibodies. (**A**) Representative pseudocolor plots showing, for MR1/5-OP-RU tetramer^{+}Vα7.2^{+} T cells (top row) or for MR1/5-OP-RU tetramer-Vα7.2^{+} T cells (bottom row), CD4 and CD8α staining on total cells (left plots for each donor group) and CD8α and CD8β staining on CD8α^{+} cells (right plots for each donor group).

Ryu A, Clagett BM, Freeman ML. Inflammation and Microbial Translocation Correlate with Reduced MAIT Cells in People with HIV. *Pathogens and Immunity*. 2024;10(1):19–46. doi: 10.20411/pai.v10i1.746

Numbers on plots indicate the percent of cells in the indicated gate. (**B**) Pie charts showing the proportion of MR1/5-OP-RU tetramer$^+$ Vα7.2$^+$ CD3$^+$ T cells (top row) and MR1/5-OP-RU tetramer$^-$ Vα7.2$^+$ CD3$^+$ T cells in each of the indicated subsets. Analysis of the cell subset distribution is shown in the matrix and was performed using the SPICE permutation test with 10,000 iterations. (**C**) Proportion of MR1/5-OP-RU tetramer$^-$ Vα7.2$^+$ CD3$^+$ T cells that are CD8$\alpha\alpha^+$. Lines and error bars indicate the median ± IQR. (**D**) Number of CD8$\alpha\alpha^+$ MR1/5-OP-RU tetramer$^-$ Vα7.2$^+$ CD3$^+$ T cells per μL of peripheral blood. Lines and error bars indicate the median ± IQR. (**E**) Proportion of MR1/5-OP-RU tetramer$^-$ Vα7.2$^+$ CD3$^+$ T cells that are CD4$^+$. Lines and error bars indicate the median ± IQR. (**F**) Number of CD4$^+$ MR1/5-OP-RU tetramer$^-$ Vα7.2$^+$ CD3$^+$ T cells per μL of peripheral blood. Lines and error bars indicate the median ± IQR. Statistics for **C-F** were calculated using Kruskal-Wallis test with Dunn's correction for multiple comparisons. ns, not significant; *P<0.05; ***P<0.001; ****P<0.0001.

PATHOGENS AND IMMUNITY
IN ASSOCIATION WITH CASE WESTERN RESERVE UNIVERSITY AND THE RICHARD J. FASENMYER FOUNDATION

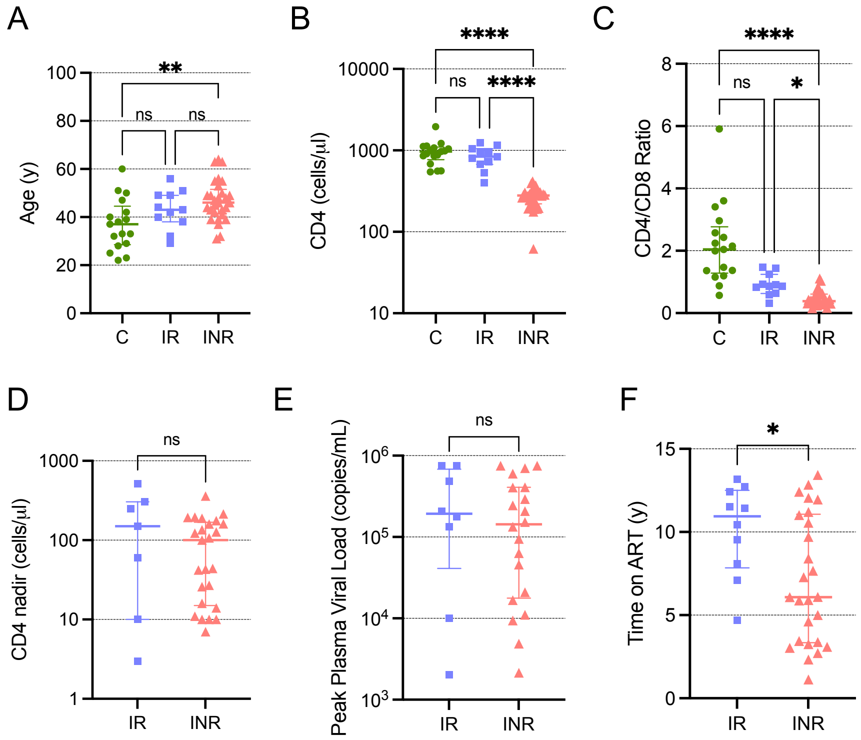

Supplementary Figure 4. Clinical comparison of Cleveland Immune Failure study donors. (**A**) Age of Cleveland Immune Failure (CLIF) study control (C, n=17), immune responder (IR, n=11), and immune non-responder (INR, n=29) donors. Lines and error bars indicate the median ± IQR. (**B,C**) Number of $CD4^+$ T cells per μL of peripheral blood (**B**) and CD4/CD8 T cell ratio (**C**) from C (n=17), IR (n=11) and INR (n=30) donors. Lines and error bars indicate the median ± IQR. (**D**) Nadir CD4 T cells per μL of peripheral blood from IR (n=7) and INR (n=25) donors. Lines and error bars indicate the median ± IQR. (**E**) Peak plasma HIV-1 RNA (viral load) copies per μL of peripheral blood from IR (n=8) and INR (n=20) donors. Lines and error bars indicate the median ± IQR. (**F**) Time on antiretroviral therapy (ART) for IR (n=10) and INR (n=26) donors. Lines and error bars indicate the median ± IQR. Statistics for **A-C** were calculated using Kruskal-Wallis test with Dunn's correction for multiple comparisons. Statistics for **D-F** were calculated using Mann-Whitney U test. ns, not significant; *P<0.05; **P<0.01; ****P<0.0001.

PATHOGENS AND IMMUNITY
IN ASSOCIATION WITH CASE WESTERN RESERVE UNIVERSITY AND THE RICHARD J. FASENMYER FOUNDATION

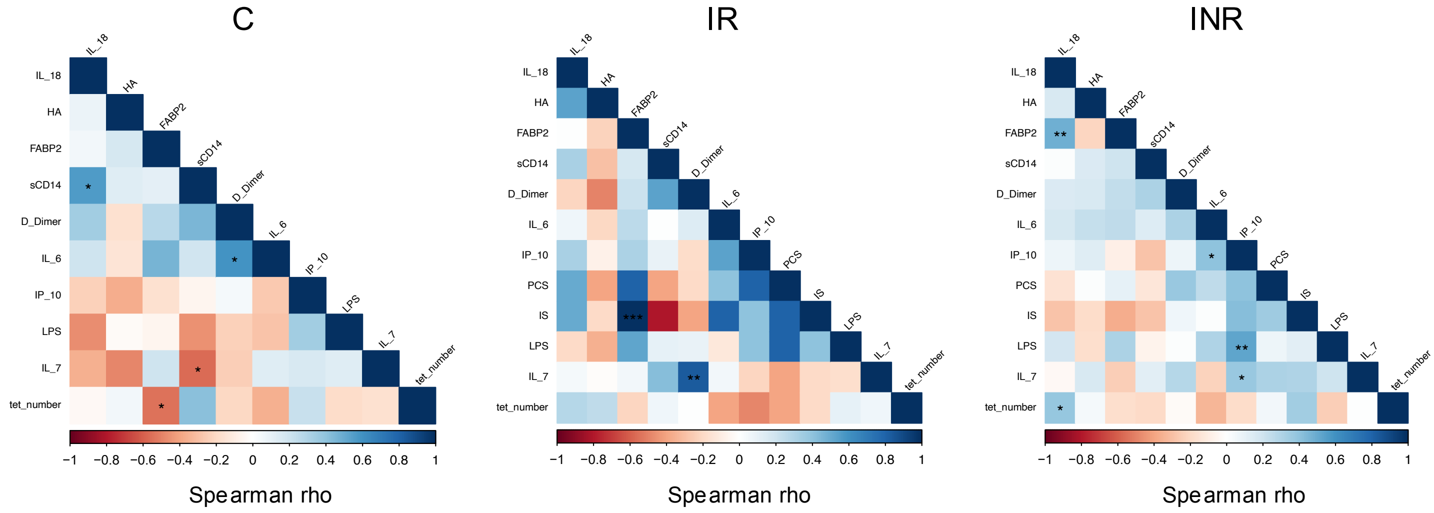

Supplementary Figure 5. Associations of soluble markers of inflammation with MAIT cell numbers and with each other within each donor group. Correlograms of MAIT cell numbers (tet_number) and soluble plasma analytes for Cleveland Immune Failure (CLIF) study control (C, n=16-17), immune responder (IR, n=4-11), and immune non-responder (INR, n=18-30) donors. Neither p-cresol sulfate (PCS) nor indoxyl sulfate (IS) were measured in plasmas of C donors. Statistics were calculated using Spearman correlation. **P*<0.05; ***P*<0.01.

Ryu A, Clagett BM, Freeman ML. Inflammation and Microbial Translocation Correlate with Reduced MAIT Cells in People with HIV. *Pathogens and Immunity*. 2024;10(1):19–46. doi: 10.20411/pai.v10i1.746

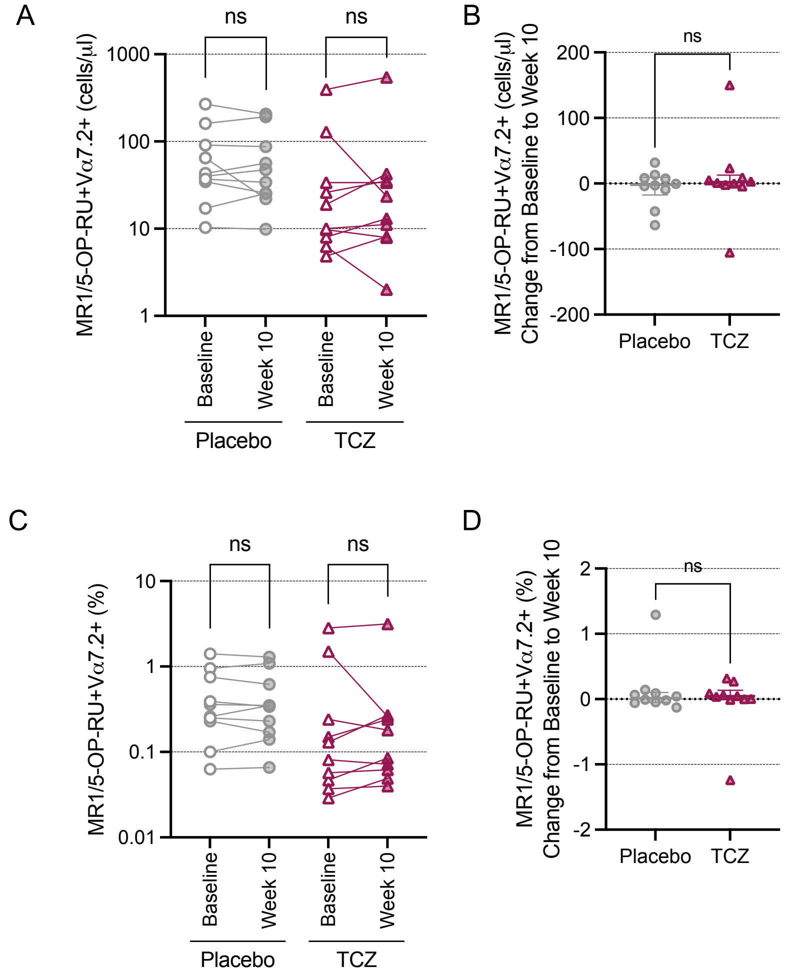

Supplementary Figure 6. There is no change in the number of MR1/5-OP-RU+ cells following inhibition of IL-6 signals *in vivo*. Cryopreserved PBMCs from NCT02049437, from baseline and at week 10 following monthly administrations (at week 0, week 4, and week 8) of tocilizumab (TCZ, n=10) or placebo control (n=10), were thawed, labeled with MR1/5-OP-RU tetramers, and stained with surface antibodies. (**A**) Number of MR1/5-OP-RU tetramer$^+$Vα7.2$^+$ CD3$^+$ T cells per μL of peripheral blood at baseline or week 10 of placebo or TCZ administration. Lines and error bars indicate the median ± IQR. (**B**) Change in the number of MR1/5-OP-RU tetramer$^+$Vα7.2$^+$ CD3$^+$ T cells per μL of peripheral blood from baseline to week 10.
(C) Proportion of CD3$^+$ T cells that are MR1/5-OP-RU$^+$Vα7.2$^+$ at baseline or week 10 of placebo or TCZ administration. Lines and error bars indicate the median ± IQR. **(D)** Change in the proportion of CD3$^+$ T cells that are MR1/5-OP-RU$^+$Vα7.2$^+$ from baseline to week 10. Statistics for **A,C** were calculated using Wilcoxon matched-pairs signed rank test and statistics for **B,D** were calculated using Mann-Whitney U test. ns, not significant.

Ryu A, Clagett BM, Freeman ML. Inflammation and Microbial Translocation Correlate with Reduced MAIT Cells in People with HIV. *Pathogens and Immunity*. 2024;10(1):19–46. doi: 10.20411/pai.v10i1.746
